# The Effect of Similarity: Non-Spatial Features Modulate Obstacle Avoidance

**DOI:** 10.1371/journal.pone.0059294

**Published:** 2013-04-29

**Authors:** Rudmer Menger, H. Chris Dijkerman, Stefan Van der Stigchel

**Affiliations:** Experimental Psychology, Helmholtz Institute, Utrecht University, Utrecht, The Netherlands; University of Muenster, Germany

## Abstract

The introduction of non-target objects into a workspace leads to temporal and spatial adjustments of reaching trajectories towards a target. If the non-target is obstructing the path of the hand towards the target, the reach is adjusted such that collision with the non-target, or obstacle, is avoided. Little is known about the influence of features which are irrelevant for the execution of the movement on avoidance movements, like color similarity between target and non-target objects. In eye movement studies the similarity of non-targets has been revealed to influence oculomotor competition. Because of the tight neural and behavioral coupling between the gaze and reaching system, our aim was to determine the contribution of similarity between target and non-target to avoidance movements. We performed 2 experiments in which participants had to reach to grasp a target object while a non-target was present in the workspace. These non-targets could be either similar or dissimilar in color to the target. The results indicate that the non-spatial feature similarity can further modify the avoidance response and therefore further modify the spatial path of the reach. Indeed, we find that dissimilar pairs have a stronger effect on reaching-to-grasp movements than similar pairs. This effect was most pronounced when the non-target was on the outside of the reaching hand, where it served as more of an obstacle to the trailing arm. We propose that the increased capture of attention by the dissimilar obstacle is responsible for the more robust avoidance response.

## Introduction

During our everyday activities we reach towards and grasp many objects. Although these objects are seldom the only items present in our direct surroundings, we are able to steer our hand toward them and evade any obstacles that are present. This ability is served by a complex system that encodes potential obstacles to an arm movement during motor planning so that they are successfully avoided during movement execution. Tresilian first described that the deviations to movements that are found after the introduction of a non-target to a workspace are the result of the preplanning of an avoidance movement to prevent collision with the non-target [1]. The nervous system is thought to modify the reaching movement in response to the presence of obstacles so as to minimize the likelihood of collision based on a preferred distance of the arm to the obstacle. The modification process itself is ostensibly subtle and precise [2]. For instance, when wrist posture is changed, the obstructing effects of a non-target object may change as well [1]. Evidence in line with this account comes from many studies in which an increase in movement time is observed when a non-target stimulus is placed in the workspace [1]–[6], suggesting that the movement is slowed down to increase spatial accuracy and avoid potential collisions. These adjustments are not a general response to the presence of non-target objects [2], on the contrary, the effect is specific to the layout of the workspace, in that non-target objects only elicit an avoidance response when the preferred distance to them is too small. For instance, Chapman and Goodale have noted that the obstacle avoidance system is sensitive to changes in obstacle size and obstacle location [7].

A number of other studies have shown that presenting non-target objects in a workspace in a position where they do not directly obstruct the reach toward a target also leads to spatial and temporal modifications of reaching-to-grasp movements [8]–[14]. In this case the non-target is regarded as a distractor. It is thought that the distractor interferes with the planning of the action toward the target object by evoking a competing response that needs to be inhibited before an accurate reach toward the target can occur [12]. This results in spatio-temporal adjustments to reach movements which are in turn modified by various features of the distractor. Among the features that have been investigated in these studies are distractor size [7] orientation [9] and location [2].

Although the explanation offered by the obstacle avoidance account can explain data more parsimoniously than the distractor interference account, the simple fact that obstacles need to be avoided to prevent a collision may not exclude other factors from further modifying the spatial path of the reach. This is true as long as additional modifications of hand movements through a workspace where obstacles need to be avoided can be revealed. So far, however, the competition between a target and a distractor has been manipulated by changing features that are also directly relevant for the execution of movement, that is, by changing features that made them more or less obstructing (e.g. size, orientation).

The aim of the current study was to investigate the effect of similarity between target and non-target on reaching behavior. More explicitly, we were interested in whether the similarity between target and non-target, defined by color, would modulate the movement trajectories of participants when they needed to avoid obstacles. Because similarity in color is not directly movement relevant, any alterations to movement trajectories are directly attributable to non-spatial features of the non-target, without a possible role for biomechanical control laws in explaining alterations to movement trajectories. Furthermore, it is known that color itself does not have an influence on the reach trajectories when controlling for chromaticity and luminance [15]. While color-matched targets and non-targets have not been studied earlier in an obstacle avoidance paradigm, there have been investigations into similarity in distractor interference setups for eye movements and hand movements. We will discuss these studies below and note some limitations.

For eye movements it has been reported that saccade trajectories deviate more away from similar distractors than dissimilar distractors [16]–[19]. This has been explained by the stronger oculomotor competition between the target and distractor when they are similar. This in turn results in a stronger top-down inhibition to resolve the competition, which leads to larger deviations away from the distractor. This line of reasoning could be applicable to hand movements as well: when there is strong competition between two similar objects, the inhibition of the automatic movement response to the ‘distracting’ object should also be strong, which should be reflected in a hand movement that veers away from a similar distractor more strongly than when a dissimilar distractor is presented (in this case a dissimilar pair of objects is associated with less inter-object competition). Of course, this hinges on the assumption that eye movements and hand movements are planned by systems that are sensitive in the same way to a given form of information (i.e. similarity information), which may not be justified. Indeed, several studies show that targets for the eye and hand are selected by independent, effector-specific, systems [20]–[22], which makes it difficult to generalize results from eye movement studies to hand movement studies.

Empirical evidence for the effect of target-distractor similarity in prehension [9] indicated that when the features of target and distractor are different, the distractor has a greater impact on prehension parameters. It is interesting to note that the features with which similarity in their study was manipulated were size and orientation. Therefore, any effect of similarity could possibly be attributable to an avoidance response to the physical properties of the non-targets instead of increased competition between target and non-target caused by those properties of the non-target. As mentioned earlier, it would therefore be informative to study similarity with a feature that does not affect the occupation of volumetric space, as it would afford a direct comparison with eye movement literature and not suffer from the aforementioned confound of possibly increasing obstructiveness.

In this study we asked participants to reach towards and grasp a target with a non-target present in the workspace. The non-target could be placed in different locations relative to the target. The target and non-target could be either similar or dissimilar in color, while they were identical with respect to all other features. We measured participants' reaching trajectories and extracted kinematic parameters from them. We were interested in finding a difference between similar and dissimilar conditions, because that would imply that using color to establish similarity is a relevant intervention in an obstacle avoidance paradigm. This would point out that non-spatial visual properties that have been found to modulate distractor effects can also modulate obstacle avoidance maneuvers.

## Experiment 1

In this experiment, we tested the reach-to-grasp behavior of participants towards a single target in the presence of either a non-target in the left hemispace or a non-target in the right hemispace. Participants reached with their right hand, so that the left hemispace non-target was on the inside of the reaching arm and the right hemispace non-target was on the outside of the reaching arm. Non-targets were either a different or the same color as the target. In addition, we observed reaching behavior to grasp a target in the absence of non-targets.

### Methods

#### Ethics Statement

The faculty's institutional review board, the WMO Advisory Committee, under the Medical Research Act issued a formal written waiver that this research project did not require approval from a Medical Ethics Review Committee. Thus, the institutional review board issued a formal written waiver for the need of ethics approval.

#### Participants

Ten (2 men, 8 women) right-handed participants contributed to this study. All participants had normal or corrected-to-normal vision and were naïve to the purpose of the study. Each participant gave written informed consent prior to the start of the experiment.

#### Materials

**Figure 1:**
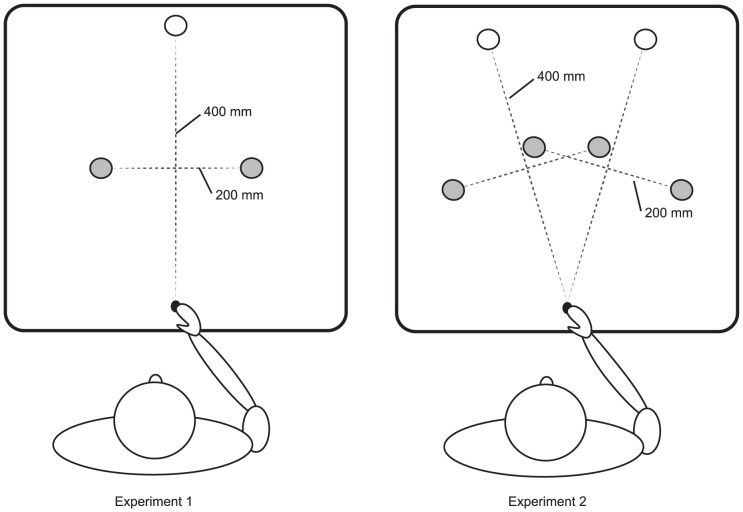
Experimental setups. Top-down view of the experimental setups. Filled circles represent possible non-target locations, while an empty circle represents the target locations. The distance between the starting location of the hand and the target location was 400 mm. Midway towards the target the non-targets were placed at 200 mm distance to the starting location. Non-targets were removed 100 mm from the midline of the workspace. Panel A represents the setup of Experiment 1 and panel B that of Experiment 2.

The participant sat in front of a 122 cm×61 cm table. This table featured a workspace of 40 cm×40 cm. In the workspace there were two fixed elements; the start button and the target. Both were shifted 3 cm rightwards from the center of the table. A virtual line connecting the middles of the start button and the target was considered as the midline of the workspace. The start button was positioned 5 cm away from the front edge of the table and the target was situated 40 cm beyond that. The non-targets were positioned at widths of 10 cm to the left and right of the midline of the workspace, that is, the non-targets were either positioned on the inside or outside of the reaching arm. Their depth was always 20 cm into the workspace (halfway in between the starting button and the target). The target was placed on a trigger that responded to when the target was lifted of it. See Figure 1, left panel, for an overview of the setup. The target and non-targets were tall wooden cylindrical objects (2.75 cm×2π×15 cm). We made two sets; one painted green and one painted red.

Participants wore PLATO LCD goggles (Translucent Technologies, Toronto, Canada), which permitted manipulation of visual feedback and participants wore MiniBird magnetic markers (Ascension Technology Corporation, Burlington, USA) which allowed kinematic tracking with a sampling rate of 100 Hz over a recording window of 3 s. These markers were placed on the tips of participants' right index finger and thumb. The cables were fixed to the participants as well as to the edge of the table with tape and elastic bands, so that participants could move their arm without restriction and without influencing the recordings.

#### Design & Procedure

There were two possible locations where objects could be placed as non-targets and a single target location. The non-targets could be either similar or dissimilar in color to the target. In addition, the target could be either red or green. The number of experimental configurations of the workspace then becomes eight, viz. two levels of similarity (Similar vs. Dissimilar) x two levels of non-target side (Inside vs. Outside) x two levels of target color (Green vs. Red). The workspace also had two configurations in which only a red or green target was presented. The total number of configurations was 10. Participants reached towards the target with each configuration 10 times, which meant we recorded a total of 100 trials. The trials were pseudo-randomized across the first and second half of the experiment, that is, participants completed five repetitions of all configurations in the first half of the experiment and in the second half of the experiment.

Participants were positioned with their midsaggital line (sternum to navel) in line with the midline of the workspace. Each trial started with an empty table and with the participants' hand on the starting button. The participants had no access to visual information at this point. The non-target and target were then carefully placed in the workspace by the experimenter, minimizing any auditory cues about the position of the target and non-target. Next, vision was returned the participant and they had to wait for an auditory ‘go’ signal after a random duration between 800–1200 ms before starting the movement. Upon the auditory signal data collection commenced. The task was to lift the target and place it back as rapidly and smoothly as possible. Once the target was lifted, the end of movement recording was triggered. After the participant had returned his or her hand to the starting button the visual masking was imposed again and the experimenter cleared the table for the next trial. Eye fixation was not restricted during the reaching movement.

#### Data processing

All analyses regarding the reaching trajectory were conducted on the data from the marker on the tip of the right index finger. Raw 3D data of each trial were filtered using a dual-pass Butterworth filter (2^nd^ order, 20 Hz cut-off). Velocities in each cardinal dimension (x, y, and z) were computed. Positions and velocities were used to define the beginning of the movement [23]. In this case the movement ‘started’ when a few separate conditions were met: (1) the index finger marker position needed to be within 3 mm of the starting location, (2) the index finger and thumb needed to be travelling faster than 5 mm/ms, (3) for at least 50 ms. A three dimensional velocity vector was determined by vector sum addition of speeds in each cardinal direction (x, y, and z) for prerequisite (2) and (3). Per trial a number of sample candidates would meet these criteria for the start of the movement (i.e. close enough to the starting button and travelling faster than the minimal speed for the given duration). A continuous function then expressed which of the samples was actually closest to the threshold of the given minimal speed: F_v_ = 1−v/v_min_. This particular sample would then be chosen as the start of the movement. Trials were rejected for the following reasons: the reach never exceeded the minimum velocity (reported above), the reach was initiated before the starting cue was given, the reach did not end within the recording window (3 s), or because of unforeseen recording errors. No participants had more than 10% of movements rejected, which was the maximum of allowed rejections.

Reach trajectories were normalized to have the same origin in a 3D Cartesian coordinate system and to have the same number of position measurements. To this end, cubic spline interpolation was used.

#### Analysis

For each participant, all the dependent measures were computed for every trial and then averaged for each of the 10 configurations. Difference scores were computed for every kinematic parameter by subtracting experimental and control conditions from each other. We used the following kinematic parameters: x-deviation at the moment the hand passes the obstacle (in mm) [7], movement duration (in milliseconds), reaction time (in milliseconds), and the initial direction of the movement (slant of the movement vector with respect to the midsaggital line in degrees after 150 ms, with clockwise as positive and midsaggital as 0). We have chosen to define this last measure as an angle instead of a distance because it allows for a more direct comparison between this study and the results from eye movement literature [16]–[19] (regarding similarity) in which angular outcome measures are used regularly.

All difference scores were subjected to a repeated measures analysis of variance having two levels of similarity (Similar vs. Dissimilar), two levels of non-target location (Outside vs. Inside) with respect to the reaching arm and two levels of target color (Red vs. Green). Interaction effects were further explored using post-hoc paired t-test analyses with Bonferroni's correction for significance.

### Results

#### Deviation at Passing

**Figure 2:**
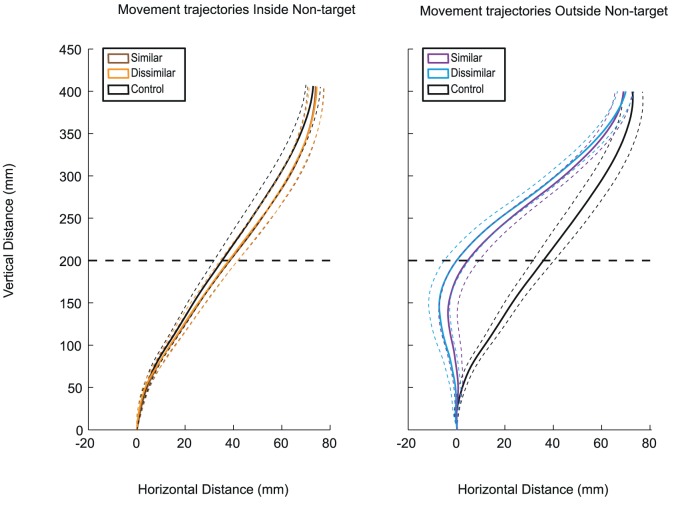
Average movement trajectories across participants for Experiment 1. Average movement trajectories across participants in the x, y plane. The left panel shows the hand trajectories when the non-target was on the inside of the hand, while the right panel shows the same for an outside non-target (with reference to the participant's reaching hand). The solid lines indicate average trajectories in the experimental and control conditions. The dashed lines represent between subjects movement error (SEM) around those trajectories. Color conditions (R and G) were collapsed into a single similar (RR and GG) or dissimilar trajectory (RG and GR), indicated by brown and purple (for the inside non-target) and orange and blue (for the outside non-target) lines respectively. The control condition is plotted in black. The horizontal dashed lines indicate the planes at which deviation at passing was measured.

There was a main effect of similarity, *F* (_1, 9_) = 13.09, *p*<.01, indicating that the mean deviation at passing for dissimilar pairs (*M* = −10.3 mm, *SD* = 1.05) was significantly greater than that of similar pairs (*M* = −9.36 mm, *SD* = 1.01). Ostensibly, participants deviated more away from a dissimilar non-target than from a similar non-target. The main effect of non-target side gave an *F* ratio of *F* (_1, 9_) = 72.62, *p*<.001. This indicates that the mean deviation at passing for inside non-targets was significantly different from the outside non-targets (compare panels A & B of Fig. 2; the reaching movement deviates less from the inside non-targets than from outside non-targets, respectively). There was no main effect of color. The interaction effect between similarity and non-target side was significant, *F* (_1, 9_) = 7.69, *p*<.05. Post-hoc analyses using Bonferroni's criterion for significance indicated that participants deviated significantly more away from the dissimilar outside non-target than the similar outside non-target, *t* (_9_) = 2.83, *p*<.025, while the difference in deviation at passing between similar and dissimilar non-target on the inside of the arm was not significant.

A separate analysis performed on conditions with inside non-targets alone showed no significant departure from control conditions.

#### Initial Direction

Analysis revealed that there were no significant effects of any factor on initial direction. We discovered two trends towards significance, however, one for non-target side with *p* = .053 and one for the interaction between similarity with non-target side, *p* = .051. This is in line with the effects described above.

#### Movement Time

None of the factors were significant for movement time.

#### Reaction Time

The analysis showed no significant main effects for reaction time. There was, however, one significant interaction effect between similarity and non-target side, *F* (_1, 9_) = 11.2, *p* = .01. Further investigation using Bonferroni-corrected t-tests revealed that participants reacted significantly slower, t (_9_) = −2.39, *p*<.025, in the dissimilar conditions (*M* = 351 ms, *SD* = 47) than in the similar conditions (*M* = 333 ms, *SD* = 53) when the non-target was presented on the outside of the arm (right side of the workspace), whereas there was no significant difference in reaction time between the different similarity conditions when non-targets were presented on the inside of the arm (left side of the workspace).

Taken together, these parameters imply that the movement trajectory as a whole was affected by similarity, as can be seen in Figure 2, at least as long as the non-target is on the outside of the arm.

### Discussion

The purpose of this experiment was to uncover the role of color-defined similarity between target and non-target by investigating hand reaching trajectories in an obstacle avoidance paradigm. We hypothesized that similarity of target and non-target would be reflected in differences in movement trajectories because of analogous results from previous research into eye and hand movements with the distractor interference paradigm. The results support this hypothesis as the trajectories of the hand are different when target and non-target have the same color compared to when they have different colors. This effect is a replication of Kritikos and colleagues [9] who found an analogous effect for size and orientation and Ludwig and Gilchrist [16] who report a similarity effect of visually presented targets and distractors for eye movements. This ‘similarity’ effect thus extends to color-defined similarity in physical objects and we now have support for an additional modifier of the hand trajectory in obstacle avoidance beyond the avoidance of a collision.

There is a further distinction that we can make with regard to the direction for the similarity effect; our results indicate that a dissimilar pair causes more deviation away than a similar pair. This is in line with Kritikos and colleagues [9] results but not with the results of Ludwig and Gilchrist [16]. We refer to the general discussion for a detailed treatment of the direction of the similarity effect.

The results also indicate an effect of non-target location on reaching trajectories. This could have been because the right side non-target (on the ‘outside of the arm’) is actually more of an obstacle to the trailing lower arm than the left side non-target. All reaches were made with the right hand, so the enlarged movement trajectories ‘around’ the right side non-target may reflect the constraints placed on the movement of the lower arm which forced it in a direction away from the non-target to avoid collision–and moving the hand with it (for a detailed discussion see [24]).

Of particular interest is the interaction we found between non-target side and similarity between target and non-target. We can discount obstacle avoidance as a cause, because similarity was manipulated using color which is a feature that does not necessitate movement adaptations in itself, contrary to a feature like non-target orientation, which–if manipulated–leads to a different physical layout. Because in our experiment the physical location of the non-target was constant and the starting posture was controlled, the difference between movements with similar and dissimilar pairs present in the workspace cannot be due to biomechanical considerations. The effect is therefore due to a subtle bias caused by the relation between non-target and target. It may be that a particular level of obstruction by the non-target is required before this effect of similarity can affect the behavior of participants, which could explain why we find the effect of similarity only on the right side of the workspace where more robust avoidance maneuvers were observed.

## Experiment 2

In Experiment 1 we showed that a non-target of a different color than the target gave rise to larger avoidance effects in the reach-to-grasp movement than a non-target with the same color as the target. This effect appeared to be driven by the right non-target position. To account for the dominant effect of the right non-target position we proposed that this non-target position may have been more obstructive to the trailing arm. Furthermore, we speculated that the requirement of more robust avoidance maneuvers in the right non-target condition may have made the similarity effect more readily detectable. In that case, the detection of the effect of similarity is facilitated by the biomechanical constraints placed on the movement system. It is worthwhile to investigate whether the similarity effect can be replicated under slightly different biomechanical constraints so as to exclude an effect of task ecology. We performed Experiment 2 to check whether non-spatial features of the obstructing non-targets can that still further modify avoidance responses when reaches are made in different directions (other than straight ahead) into either the left or right hemispace.

To this end we modified the setup of Experiment 1 such that participants had to reach to grasp towards a target that could be present in one of two possible locations that were 30 degrees apart. Targets were presented in isolation and in the presence of non-targets that were to the left or right of the target. Non-targets were similar or dissimilar to the target with regard to their color. In half the trials the non-target was on the outside of the reaching arm, while the non-target was on the inside of the reaching arm in the other half of the trials.

To recap, we expected that non-targets that were dissimilar to the target would evoke larger avoidance responses than similar ones. In addition, any such (dis)similarity effect was expected to be confined to situations where the non-target was on the outside of the reaching hand, irrespective of the location of the target.

### Methods

The method for Experiment 2 was similar to that of Experiment 1; as such only the differences in methodology are reported here.

#### Ethics Statement

The faculty's institutional review board, the WMO Advisory Committee, under the Medical Research Act issued a formal written waiver that this research project did not require approval from a Medical Ethics Review Committee. Thus, the institutional review board issued a formal written waiver for the need of ethics approval.

#### Participants

Ten right-handed participants volunteered to participate in this study (4 men, 6 women). All had normal or corrected-to-normal vision. No participants were excluded based on their failure to meet our mistrial criteria. Each participant gave written informed consent prior to the start of the experiment.

#### Materials

The setup of Experiment 1 was rotated 15 degrees clockwise and counterclockwise to create two new target and four new non-target locations. In the workspace there were two fixed elements; the start button and a target at one of two possible locations. Virtual lines connecting the middles of the targets with the start button were considered as the midlines of two (overlapping) workspaces. The start button was positioned 5 cm from the front edge of the table and the target buttons were situated 40 cm along ‘their’ midlines beyond that. The non-targets could be positioned at widths of 10 cm to the left and right of the midlines of the workspaces. Because the participants always used their right hand for grasping, the ‘left’ non-targets were on the inside of the reaching arm irrespective of target location, whereas the ‘right’ non-targets were on the outside of the reaching arm irrespective of target location. Non-target depth was always halfway from the start button to the target along the midline from starting button to target location. The targets were placed on triggers that responded to the removal of the targets. See Figure 1, right panel, for an overview of the setup.

#### Design & Procedure

There were four possible locations where objects were placed as non-targets and two target locations where targets were placed. Per target location there were two possible non-target locations. Furthermore, the non-targets could be either similar or dissimilar in color to the target. In addition, the target could be either red or green. The number of experimental configurations of the workspaces was 16, viz. two levels of similarity between targets and non-targets (Similar vs. Dissimilar) x two levels of target side (Left vs. Right) x two levels of non-target side (Inside vs. Outside) x two levels of target color (Green vs. Red). The experiment also had four control conditions in which only a red or green target was presented at either target location. The total number of configurations was 20. Each configuration was executed eight times, to a total of 160 trials. Trials were randomized across the first and second half of the experiment.

#### Analysis

For each participant, all the dependent measures were computed for every trial and then averaged for each of the 20 configurations. We calculated the spatial measures with respect to the midline of the workspace associated with a particular condition in which the movement was performed. This means that the scores within this experiment and between Experiment 1 and 2 are directly comparable. Difference scores were computed for every kinematic parameter by subtracting experimental and control conditions from each other. We used the following kinematic parameters: x-deviation at the moment the hand passes the obstacle (in mm), movement duration (in milliseconds), reaction time (in milliseconds), and the initial direction of the movement (slant of the movement vector with respect to the midsaggital line in degrees after 150 ms).

All difference scores were subjected to a repeated measures analysis of variance having two levels of similarity (Similar vs. Dissimilar), two levels of target location (Left vs. Right), two levels of non-target location (Outside vs. Inside) and two levels of target color (Red vs. Green). Interaction effects were further explored using post-hoc paired t-test analyses with Bonferroni's correction for significance.

### Results

#### Deviation at passing

There was no main effect of similarity. For non-target side we found a main effect, *F* (_1, 9_) = 449.2, *p*<.001. This indicated that participants deviated significantly more away at passing an outside non-target (*M* = −47.1 mm, *SD* = 2.73) than an inside non-target (*M* = 1.51, *SD* = 1.09). In addition, target side had a significant main effect on deviation at passing, *F* (_1, 9_) = 5.70, *p*<.05. This means that mean deviation at passing when reaching for the right side target (*M* = −25.6 mm, *SD* = 1.89) was significantly greater than that of a left side target (*M* = −20.1 mm, *SD*  = 2.24). A main effect of color was not found.

We observed an interaction effect of similarity with target side *F* (_1, 9_) = 7.80, *p*<.025. Post-hoc analyses using Bonferroni's criterion for significance indicated that average deviation at passing was significantly more away in the dissimilar pair with the right target condition (*M* = −25.6 mm, *SD* = 1.95) than in the similar pair with the right target condition (*M* = −20.1 mm, *SD* = 2.30), *t* (_9_) = 2.55, *p*<.025, whereas if the reach was towards the left target then the similarity conditions did not differ significantly from each other. Furthermore, we determined that there was a three-way interaction effect of similarity with target side and non-target side *F* (_1, 9_) = 8.08, *p*<.025. Post-hoc testing indicated that reaches veered more away from a dissimilar ‘outside’ non-target when the target was on the right side of the workspace than from a similar non-target, *t* (_9_) = 4.75, *p*<.001. This effect was not apparent when the participants reached towards the left target or when non-targets were on the inside of the reaching arm (all *p*'s>.05). Again, it appears that the effect of similarity, or rather dissimilarity, is confined to a specific situation.

This is further substantiated by a separate comparison of inside non-targets with the control condition. Although there was no overall effect of inside non-targets on the hand movement, further (Bonferroni-corrected) testing revealed that there was one ‘inside’ condition that showed a significant departure from a reach toward a target in isolation: when the dissimilar non-target was present and participants reached toward the right hemispace target their reaching trajectories differed significantly from control conditions, *t* (_9_) = −4.27, *p*<.005.

#### Initial Direction

We found a main effect of similarity on initial movement direction, *F* (_1, 9_) = 6.10, *p*<.05. Target side also had a main effect on initial direction, *F* (_1, 9_) = 106.9, *p*<.001. Non-target side did not have a significant effect on initial direction, although it did display a trend towards significance. We found an interaction of similarity with target side, *F* (_1, 9_) = 8.0, *p*<.025 along similar lines as the interactions reported above, and an interaction between target side and non-target side *F* (_1, 9_) = 133, *p*<.001.

**Figure 3:**
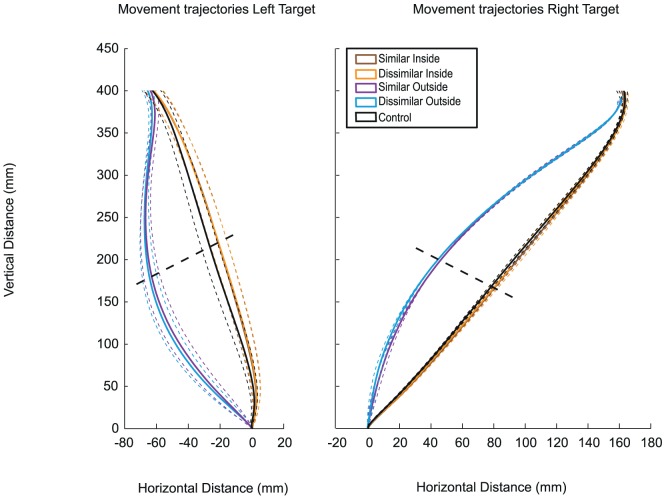
Average movement trajectories across participants for Experiment 2. Average movement trajectories across participants in the x, y plane. The left panel shows the hand trajectories towards the target on the left, while the right panel shows the hand paths towards a right side target (with reference to the participant). Each panel contains trajectories towards the target with inside and outside non-targets. The solid lines indicate average trajectories in the experimental and control conditions. The dashed lines represent between subjects movement error (SEM) around those trajectories. Color conditions (R and G) were collapsed into a single similar (RR and GG) or dissimilar trajectory (RG and GR), indicated by brown and purple (for the inside non-target) and orange and blue (for the outside non-target) lines respectively. The control condition is plotted in black. Please note the difference in scaling of the x axis compared to the previous figure. The oblique dashed lines indicate the planes at which deviation at passing was measured.

As can be seen in Figure 3 the different setups yielded quite distinct movement patterns. Please note that the x-axis was scaled differently because for grasping the right target object the index finger had to move ‘around’ it while the index finger could stay on the inside of the left target object for a successful grasp. Therefore, the horizontal distance for the movements to the right target (Figure 3 right panel) was larger (due to the added width of the object and the finger) than the distance to the left (Figure 3 left panel).

The trajectories that resulted from movements with non-target present on the outside of the reaching arm were distinct from the control conditions, whereas non-targets on the inside of the arm prompted responses that were closer to a reach toward a single target. The effects of the non-targets on the movement trajectories were therefore strongest for the outside non-targets which was reflected in the interaction effect mentioned above. In addition, the similarity effects appear to be strongest in these cases as well, see also Figure 3.

#### Reaction time

We found no significant differences between conditions for reaction time.

#### Movement Time

We found no significant differences between conditions for movement time.

### Discussion

In Experiment 2, participants were instructed to reach for and pick up a target placed 15 degrees to left and 15 degrees to the right of their midsaggital plane. The target was presented alone or accompanied by a non-target. The non-target was either similar or dissimilar in color to the target and was placed halfway between the start and target location to either the left or to the right of the reaching arm, thereby putting it on either the outside or inside of the reaching arm.

Our results indicate that non-target location had an influence on reaching trajectories, that is, non-target location influenced reaching behavior when the non-target was in a specific location, namely, to the right or on the outside of the reaching arm. Conversely, we found no systematic evidence to support avoidance effects by non-targets to the left or on the inside of the reaching hand. It is interesting to note that this ‘right side’ effect exists when a target is to the left as well as to the right of the starting hand location. More importantly, our results indicate that similarity influences reaching behavior. Key kinematic parameters were influenced by our manipulation of similarity using color. As such, we have replicated the similarity effect of Experiment 1 for additional reaching directions.

Interestingly, the similarity effect again appears to be driven by a particular configuration of the workspace; when the non-target is on the outside of the reaching hand and the target is located on the right the effect is most explicit. In this situation the non-target is located in a position that more easily allows collisions, not necessarily with the hand in isolation, but rather with the lower arm, which might explain why a bias was detected at all. Perhaps a difference due to the manipulation of non-spatial properties of the non-target is only detectable in situations where a particular level of obstruction is generated by the non-target.

## General Discussion

In two experiments, participants reached for and grasped a target that was either presented alone or in the presence of a non-target. Our main manipulation was the similarity of target and non-target. We used color to set the similarity of the target and non-target. Our secondary manipulation was the location of the non-target, which was presented either on the inside or on the outside of the reaching arm. Our aim was to investigate the effects of a non-physical, or non-spatial, feature of the non-target on the avoidance trajectory of the hand around it when it was being guided toward a to-be-picked-up target.

The results indicate that similarity is used to prompt spatial modifications to reaching trajectories. That is, dissimilar targets and non-targets appear to prompt reach trajectories that deviate further away from the non-target than similar non-targets and targets. This stronger avoidance effect seems robust for different reaching directions. In addition, the effect appears to be most pronounced when the non-target is placed on the ‘outside’ of the reaching arm.

Our results imply that using color to manipulate target-non-target similarity is a way to further manipulate the avoidance responses around non-targets. It is important to note that this effect should not be confused with that caused by a physical property of the non-target, as for the avoidance movement around a physical obstacle the color of that obstacle should be irrelevant. Therefore, our results support the idea that avoidance movements may also be further modified by non-spatial features of the non-target (in addition to very useful spatial features such as its location). This is in line with previous empirical evidence on similarity between target and non-target [9]. These authors found that dissimilarity in size and in orientation of target and distractor caused more interference on reaching trajectories. These authors speculated that their results were indications of a more general principle: ‘it is not so much size or orientation that is crucial, but rather whether the non-target is the same as or different from the target’ (p. 148, [13]). Our findings that demonstrate the same effect of similarity, although defined by the non-spatial feature color and in an explicit obstacle avoidance paradigm, seem to subscribe to this.

Our study indicated an opposite direction of the effect of similarity on hand movements to that reported for eye movement studies [16], [18], [19]; indeed, these authors find that when the visual target and the visual distractor are similar that eye trajectories deviate more away, whereas our results show stronger avoidance responses for dissimilar non-targets. One possible explanation for this discrepancy could be that eye movements and hand movements are not governed by a common attentional mechanism, which is one of the two current interpretations for the relation between eye and hand movements [20]–[22]. The other interpretation favors the idea that targets for the eye and the hand are selected by a common mechanism [25], [26]. If that would have been the case, then the departure of our results from the general tendency in eye movement literature that similar non-targets are associated with more deviation, would be quite interesting. This study, however, did not aim to distinguish between the two interpretations. Nevertheless, because the modification of avoidance responses by similarity information is different from the modification of eye movement by the same information, our data seem to point to effector-specific control systems.

Our results indicate a difference in movement trajectories between color similar and dissimilar pairs. The similarity in color between target and non-target is a feature that should not increase or decrease the likelihood of collision and should therefore not affect avoidance movements in that similarity is not a feature that is directly relevant for movement. Avoidance of a collision is still the main drive behind the modification of the spatial path of the reach through the workspace when non-targets are present. However, the avoidance response can be further modified as is evident from our similarity effect. This is based on the results from the configurations in two experiments where the non-target was on the outside of the reaching arm. As stated earlier, we hold that in these configurations the obstruction offered by the non-targets is higher and that the subtle biasing influence of color-defined similarity becomes more readily apparent. Although this statistical interpretation of our data is viable, we speculate that the enhanced influence of ‘distracting’ features of the non-target, such as its similarity, may lead to the observed biases in the movement trajectories. Simply put, any obstacle that needs to be avoided needs to be noticed first. As such, if different degrees of noticing or attentional capture are assumed, then our results may imply that hand movements veer away differently from similar and dissimilar non-targets because these types of non-targets capture different degrees of attention. Our results indicate that dissimilar non-targets may compete more for attentional resources with the targets then similar non-targets. Following Tipper and colleagues [12], the dismissal of the irrelevant dissimilar non-targets may then require increased resources which leads to increased spatio-temporal interference as evidenced from the changes in the kinematic parameters of the movement trajectories.

There has been extensive research into the neural substrates required for obstacle avoidance [27]–[31]. It has been suggested based on this work that automatic avoidance of obstacles is a dorsal stream function. Our results add an interesting new dimension to this statement, since our results suggest that not only ‘dorsal stream’ features, such as size and orientation, are relevant to obstacle avoidance, but that ‘ventral stream’ features also play a role. In this case color was the ventral stream feature. Whether there is cross-talk between the two streams in healthy participants [32] or the dorsal stream is capable of processing color to some extent is a question beyond the scope of this paper. Furthermore, in a recent paper, evidence was brought forward that indicated that conscious processing of visual information influenced obstacle avoidance [33]. Taken together with the results of the current study, this casts doubt on the presumed automatic and subconscious nature of this ability. However, further research should investigate more effects of ventral stream features in obstacle avoidance and, if possible, in participants who have impaired processing of said features.
